# Life Course Nature Exposure and Mental Health Outcomes: A Systematic Review and Future Directions

**DOI:** 10.3390/ijerph18105146

**Published:** 2021-05-12

**Authors:** Dongying Li, Tess Menotti, Yizhen Ding, Nancy M. Wells

**Affiliations:** 1 Department of Landscape Architecture and Urban Planning, Texas A&M University, College Station, TX 77843, USA; tmenotti@tamu.edu (T.M.); yizhend2@tamu.edu (Y.D.); 2 Department of Design and Environmental Analysis, College of Human Ecology, Cornell University, Ithaca, NY 14853, USA; nmw2@cornell.edu

**Keywords:** life course, nature, greenness, exposure to nature, mental health, systematic review, early life, later life

## Abstract

Recently, an emerging body of literature has examined the relationships between early life nature exposure and mental health in later life; however, no critical synthesis yet exists regarding the extent and strength of these relationships. This study presents the first systematic review of studies in this growing area. Following the PRISMA framework, we searched six databases (i.e., Scopus, Web of Science, MEDLINE, Embase, PsycINFO, and CINAHL); conducted identification, screening, eligibility, and inclusion analyses; and identified a final set of 29 articles. The review set comprises primarily longitudinal studies, with several cross-sectional studies using retrospective measures of childhood nature exposure. The majority of included studies were published between 2016 and 2020 and conducted in Europe and North America. Five domains of mental health outcomes are associated with early-life nature exposure: incidence of mental disorders, psychiatric symptoms and emotions, conduct problems in children, cognitive function, and subjective well-being. The evidence lends support to an overall beneficial role of early nature exposure on mental health, although inconsistencies are reported. Taken together, the evidence does not suggest that exposure at any given life stage is more saliently associated with mental health outcomes than at others. We discuss the validity concerns and methodological remedies and offer directions for future research.

## 1. Introduction

Nature plays a critical role in human mental health and well-being. Cross-sectional research has associated nearby nature with a variety of mental health benefits for children [1,2,3], adolescents [4,5], adults [6,7], and older adults [8,9]. Reviews that synthesized evidence from multiple studies have also confirmed that benefits of nature may occur at various life stages [10,11,12]. However, such cross-sectional evidence does not imply a long-lasting influence over the course of a life. 

In the last decade, interest has developed in studying the effects of nature within a life course framework. The life course perspective [13,14,15] emphasizes the long-term effects of social and environmental factors during one or more life stages (e.g., gestation, childhood, adolescence, adulthood, and later adulthood) on development and health in later life. The life course approach acknowledges that a person’s health conditions are shaped by past and present experiences, which can set or change a person’s life trajectory toward a certain outcome [16,17]. Childhood interactions with nature can impact life-long trajectories regarding an individual’s relation to natural environments. For example, participation in nature-related activities during childhood is related to having a pro-environmental attitude and preference for and likelihood of visiting nature during adulthood [16,18]. 

Integration of the life course perspective into nature and health research provides a unique angle in disease prevention and health promotion. Instead of focusing on health as merely “absence of disease”, this view aligns with the holistic definition of health as “a state of complete physical, mental, and social well-being” [19] and recognizes that the benefits of nature experiences during certain stages may last longer [20] and change health conditions in later life more profoundly than what is typically thought of as the effects of “a dose of nature”. Instead of assuming that a person is healthy until disease occurs, this framework rests on the premise that an individual’s health trajectory may be determined early in life, supporting timely identification and interventions that would be most effective in non-communicable diseases such as mental disorders [21].

In analyzing the long-term benefits of nature exposure, to date most attention has been paid to knowledge, awareness, attitude, and behavioral outcomes. Studies focused on nature’s relation to mental health have surged only in the last five to six years [22,23,24,25]. Moreover, there has not yet been a systematic review that synthesizes and evaluates how early-life exposure to nature is associated with later-life mental health outcomes. A timely systematic review could identify trends, gaps, and challenges in this rapidly growing area; inform future research; and guide health care delivery, environmental planning, and policy development. 

Therefore, the aim of this work is to critically assess empirical observational studies reporting relationships between early-life exposure to nature and later-life mental health outcomes across all age ranges. The central hypothesis is that higher levels of early-life exposure to nature are associated with better later-life mental health. We conduct a systematic literature review to uncover evidence, investigate the consistency of findings, and determine needs for future research. In addition to testing the cumulative evidence regarding the central hypothesis, we ask four specific questions:

1. What types of mental health benefits are associated with early-life nature exposure?
2. How strong and consistent is the evidence relating to associations between life course nature exposure and mental health?
3. To what degree does the current evidence support a critical period or time-variant effect of nature on mental health?
4. What theoretical and methodological challenges and unanswered questions remain for future studies?

## 2. Materials and Methods

### 2.1. Search Strategy

We developed our review protocol based on the preferred reporting items for systematic reviews and meta-analyses (PRISMA) [26], which outlines standards for upholding rigor in systematic reviews. A four-step procedure was applied: identification, screening, eligibility, and inclusion. The reviewed material was obtained through a literature search performed in June 2020 using six databases: Scopus, Web of Science, MEDLINE, Embase, PsycINFO, and CINAHL. These databases were selected for complete coverage of four fields of study: environment, psychology, public health, and medicine. To ensure this review included all eligible articles, a publication date range was not set. As such, the searches yielded articles published from as early as the databases have records until June 2020.

Based on our research questions, two concept domains were used to construct the search syntax. The search rules specified that the topic fields of the article (i.e., title, author keywords, or abstract) needed to match at least one keyword from domain 1 and another from domain 2. Domain 1 related to monitoring the study population using a life course perspective, including keywords such as “life course”, “life-stage”, “life-trajectory”, “lifelong”, “grow-up”, later-life”, and “life-experience”. Domain 2 covered nature exposure, including keywords such as “green space”, “greenness”, “urban nature”, “exposure to nature”, “park”, “tree cover”, “garden”, “urban forest”, “vegetation”, “landscape”, and “ecosystem”. We used wildcards for all six databases to account for varying forms of the keywords. We did not specify a concept domain for mental health-related outcomes, because mental health is a broad umbrella that covers a wide range of terms. Since our research objective was to identify the types of mental health benefits associated with life-long exposure to nature, any predefined enunciation would confer bias; therefore, we allowed the categories to arise from the data. For an example syntax, see Supplementary Material I. 

Citation chaining including forward and backward searches [27] using eligible articles was also performed. In addition, we screened one curated database, the Children and Nature Network (C&NN) Research Library [28], to ensure eligible articles were included in this review. 

### 2.2. Inclusion Criteria

After combining the records from the six databases and removing duplicates, we conducted the screening and eligibility procedures by reviewing the title, abstract, and full text sequentially. For every record, two researchers performed the tasks independently; disagreements were resolved through discussion and with the input of a third researcher when necessary. 

Studies were included in the review if they:

- Reported original empirical research published in a peer-reviewed journal;
- Were written in English;
- Included a measure of nature or green space as exposure (or independent variable). We included both objectively-measured and self-reported green space, regardless of whether the measure was related to nearby nature close to home or other types of visits to or use of green space. Both quantity and quality measures were considered eligible;
- Reported at least one measure of mental health as the outcome, including psychological, affective, and cognitive measures;
- Examined the effects of early-life nature exposure or exposure trajectories on later-life mental health, or involved one of the three important aspects of the life course approach: critical periods, pathways, and accumulation [17,29];
- Performed an inferential statistical test to examine the relationship between nature exposure and mental health outcome.

The main reasons for article exclusion included not involving a life course perspective (e.g., cross-sectional or experimental studies on short-term effects), qualitative studies on the same topic, outcome variable not relevant, or exposure variable not relevant. Specifically, concerning outcome relevance, we excluded studies that reported outcomes other than mental health conditions: for example, environmental knowledge, preference, attitude, or environmental behavior; physical activity, sedentary behavior, or sleep–diet patterns; and frequency of visits to greenness areas or participation in nature or outdoor leisure activities. Although these factors can be correlated with mental health conditions, we restricted our review set to articles that reported inferential statistics between nature exposure and at least one mental health outcome. 

### 2.3. Assessment of Study Bias

The risk of bias of the included studies was assessed (see Supplementary Material II) using the Study Quality Assessment Tool [30] developed by the National Heart, Lung, and Blood Institute (NHLBI). Compared to other widely-used quality assessment tools (e.g., the Joanna Briggs Institute Critical Appraisal tools), this scale can simultaneously evaluate observational cohort and cross-sectional studies; it also features evaluation items that are appropriate for the types of studies that involve exposure and health outcomes. This scale consists of 14 questions, each of which was rated yes, no, or not applicable or not reported. Each study was assessed by two researchers independently, and a consensus was reached through discussion whenever discrepancies occurred. Based on the count of the items receiving “yes” ratings, we further classified study quality as poor, fair, or good. Notably, because the studies demonstrated high heterogeneity in study design and time span, we consider the evaluation outcomes somewhat arbitrary. As such, we used the quality assessment to evaluate potential biases of the studies but not to exclude any study. Instead, we discuss methodological challenges in the Discussion Section. 

### 2.4. Data Extraction and Synthesis

A standard form was developed and used to extract and tabulate information from the included studies. Specifically, the extracted information related to basic study characteristics, population, study design, exposure, outcome, statistical inference, and statistical results. Three researchers performed the data extraction independently, with a 30% record overlap for confirmation and reviewing agreement. Study characteristics included the field of the first author, field of the journal, geography of the study, and setting (urbanicity) of the study. Study population included population characteristics, age (range) of exposure, age (range) of outcome measurement, sample size, and sampling procedure. Study design included type of study (cross-sectional, longitudinal, or case–control), informant, survey mode, and waves or cohorts for longitudinal studies, as well as name of dataset if using data from a larger project. Exposure included the type of nature exposure examined, measure of nature exposure, spatial unit of analysis, and spatial resolution of the measurement. The outcome category included the type of mental health outcome and the measure. The statistical inference field captured the type of statistical analysis conducted to examine the nature–mental health relationship and the covariates included in the model. The statistical results field extracted the coefficient, confidence interval, and level of significance between each nature–mental health pair; or in more complex cases, the interactions or other terms that involved a nature–mental health relationship. Due to high heterogeneity of study design, exposure and outcome measures, and age span across the studies, a meta-analysis was not possible. As such, data were synthesized through descriptive statistics, cross tabulation, plotting, and visualization. Confidence in cumulative evidence was evaluated based on the number of studies reporting such evidence; agreement of study results; quality or bias assessment for the individual studies reporting such results; and population, geographic, and measurement homogeneity. Endnote and Microsoft Excel were used to manage the data throughout the review. 

### 2.5. Protocol Registration

The protocol for this systematic review has been published in the PROSPERO International Prospective Register of Systematic Reviews (#CRD42020198342). 

## 3. Results

### 3.1. Study Characteristics

#### 3.1.1. Temporal Trends, Discipline, and Geographical Distribution

**Figure 1:**
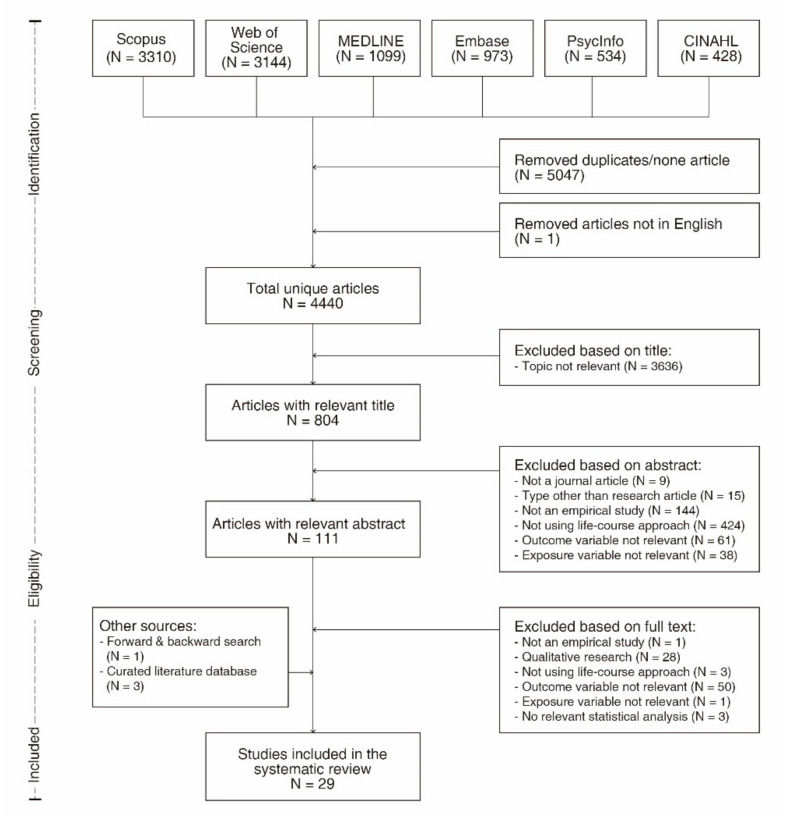
PRISMA flow diagram of the process of literature identification, screening, eligibility, and inclusion.

Our initial database search yielded a total of 9488 records (Figure 1), of which 804 were included after title screening and 111 were included after abstract screening. Upon full text review and incorporating studies from forward &backward searches and curated database screening, 29 studies were included in the final review set. The characteristics of the included studies are shown in Table 1.

**Figure 2:**
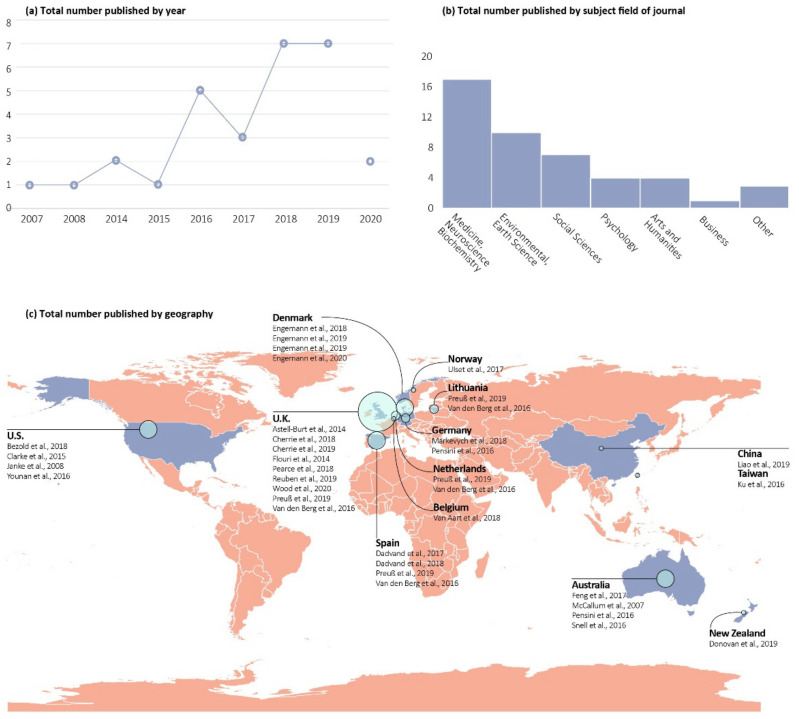
Temporal trends, subject fields, and geographical distribution of reviewed studies.

The review set demonstrated that the topic of nature and health has witnessed rapid recent growth, especially since 2015 (Figure 2a). The earliest study that met our criteria was published in 2007, although only one additional study was published from 2007 to 2014. After 2014, the body of published research grew considerably, peaking in 2018 and 2019. According to journal subject categories defined by SCImago, the reviewed articles were published in journals focused on a variety of disciplines (Figure 2b). Medicine, neuroscience, and biochemistry were the most common, followed by environmental and earth sciences, and then social sciences and psychology. In terms of the geographic distribution, the largest number of studies were from Europe (62.1%), followed by Australia and New Zealand (13.8%), North America (13.8%), Asia (6.9%), and finally cross-continent (3.4%). As the majority of the studies examined populations from Western countries, the Global South was severely underrepresented (Figure 2c). Among the articles, about one-third focused on urban settings, another third featured a mixed urban–suburban–rural setting, and the rest did not have any urbanicity-related sampling or inclusion criteria. 

#### 3.1.2. Study Design

The review set primarily comprised longitudinal (82.8%) and cross-sectional studies with a retrospective measure of childhood or lifelong nature exposure (17.2%). Informant type varied across articles, and some reported multiple types; over half (51.7%) used self-reported data, 31.0% involved parent or teacher assessments of children’s health or behavior, and another 31.0% used objective tests; medical records were also commonly used (24.1%). Several studies leveraged pre-existing datasets, especially data from carefully designed longitudinal studies (65.5%); examples included the British Household Panel Survey (BHPS) [31], the Lothian Birth Cohort 1936 [32], and the Millennium Cohort Study [33]. Despite the variety of longitudinal datasets, only one article reported a longitudinal study that was designed specifically to examine the effects of nature on health—the Positive Health Effects of the Natural Outdoor Environment in Typical Populations in Different Regions in Europe (PHENOTYPE) project. Of studies using pre-existing datasets (65.5%), most only used selected waves or populations with certain characteristics (48.3%). Another four studies (13.8%) based in Denmark used nation-wide population registries that allowed data linkage across various datasets and time points at the individual level. 

### 3.2. Measures of Lifelong Exposure to Nature

**Figure 3:**
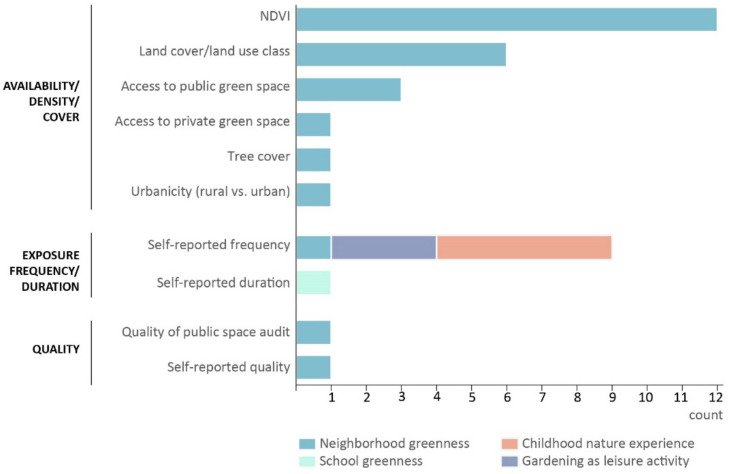
Measures of exposure to nature used in reviewed studies.

In the reviewed articles, measures of greenness exposure fell into three categories: availability/density/cover, frequency/duration, and quality (Figure 3), with availability/density/cover being the most common measure type (65.5% of studies). The most popular operationalizations of greenness exposure were the normalized difference vegetation index (NDVI) and percentages of natural land cover classes within a circular buffer around participant residential or school addresses. Buffer zone distances ranged from 100 m to 4000 m for measures including NDVI derived from multispectral satellite or aerial imagery, land cover, and access to public green space. Eleven studies performed an analysis to determine the sensitivity of the outcome to buffer distance. Findings were mixed: 45.5% of the sensitivity analyses found that the outcome was not sensitive to buffer size, while 36.4% found a greater effect with larger buffer zones (typically ~1000–3000 m radius), 9.1% found greater effect with smaller buffer zones (~100–500 m), and 9.1% found a mix of effects depending on exposure measure and outcome. Ten studies (34.5%) quantified exposure through self-reported frequency or duration of exposure to nature during childhood. Quality of greenness was measured either using neighborhood environmental audit tools [34] or by self-report [35]; however, quality measures were less common (6.9%) than the other measures.

The settings in which greenness exposure occurred generally fell into four categories (Figure 3): neighborhood (69.0%), school (3.4%), garden (10.3%), and cross-setting childhood nature experience (17.2%). In particular, studies measured childhood nature experience by asking participants to recall time spent in various natural environments, including those outside their everyday setting such as visits to national parks [36] or to the beach, mountains, or rainforests [37]. 

In many cases, the life course focus of these studies required researchers to account for the temporal dimension of exposure; this was mostly done by either calculating a cumulative measure across time, using time-varying exposure variables in longitudinal models, or including a retrospective recall item on nature experience during the entire childhood period. Most studies derived an aggregated measure (typically NDVI) over multiple exposure time periods using the mean or median [38,39,40]. Donovan et al. [41] considered minimum and maximum NDVI across two phases of each child participant’s lifetime; similarly, Younan et al. [40] examined the averages of NDVI estimates in both short-term and long-term exposure periods. Going beyond a simple mean–min–max measure, Dadvand et al. weighted NDVI based on the time lived at each residential address [42]. A second method of accounting for temporal variation in exposure was to directly use measurements from multiple time points as time-varying variables in models [42]. A third typical method involved surveying participants to recall the overall frequency of their visits to natural outdoor environments on a numeric scale [37]. This retrospective, self-reporting approach was employed in nearly all studies aiming to assess the reported childhood nature experiences of adults. 

### 3.3. Mental Health Outcomes and the Directions and Strengths of Results

**Figure 4:**
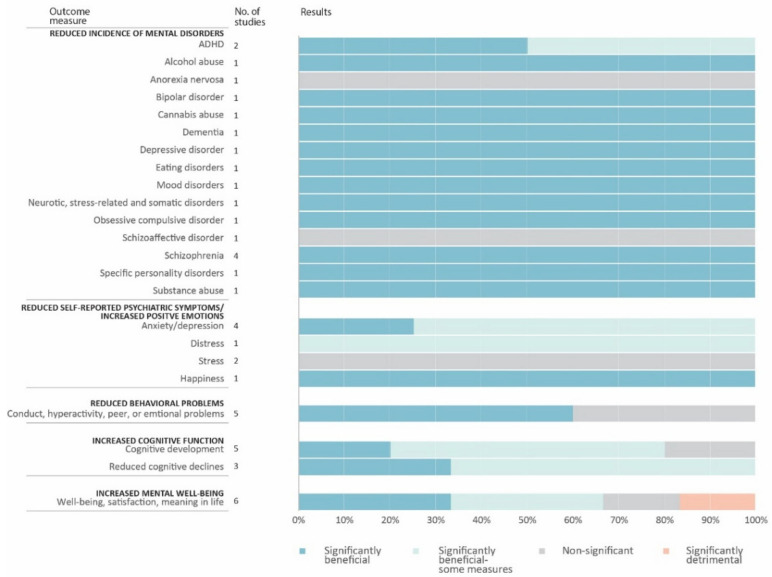
Findings related to various mental health outcomes.

Studies included in this review linked life course nature exposure with a range of outcomes, including the incidence of mental disorders, psychiatric symptoms, behavioral problems, cognitive function, and subjective well-being (SWB). While these studies investigated a variety of mental health outcomes, altogether the findings generally support a significant relationship between early nature exposure and better mental health or lower risks of mental disorders. Figure 4 illustrates the total number of articles measuring each outcome and the percentage reporting significant relationships. Of the 29 articles, 27 (93.1%) identified a significant advantageous relationship between at least one measure of nature exposure and mental health outcome. No study found a significant detrimental main effect of early life course nature exposure, and only one article (3.4%) reported an interaction effect where those who visited nature less often in childhood benefited more from undertaking more nature visits as an adult. However, as the total number of studies is small, evidence pertaining to each specific mental health outcome is limited. Among the five outcome domains, nature exposure showed the most consistent inverse association with risks of mental disorders. 

#### 3.3.1. Reduced Incidence of Mental Disorders 

The reviewed studies support a consistent negative association between childhood or life course exposure to nature and incidence of mental disorders, as indicated by diagnoses from medical records. Among the mental disorders considered, only the risks of schizophrenia and ADHD were examined in more than one study. For schizophrenia, four recent studies in Denmark demonstrated a protective role of green space exposure during childhood [38,43,44,45]. For ADHD, higher levels of neighborhood greenness were associated with decreased risk in children and adolescents. However, according to Donovan et al. [41], nature exposure between age 2 and 18 protected children against ADHD, but exposure between birth and age 2 did not. Although only reported in one study, childhood nature exposure was found to be inversely related to a wide range of mental disorders, including bipolar disorder, eating disorders, mood disorders, depressive disorder, and substance abuse [44]. For older adults, daily gardening activities predicted lower risk of dementia [46]. 

#### 3.3.2. Reduced Psychiatric Symptoms and Increased Positive Emotions 

Seven studies used psychometric instruments to evaluate psychiatric or psychological symptoms such as stress and depression. The majority reported that relations between nature and the outcome only existed for certain populations or measures. For example, evidence of a relationship between childhood nature exposure and depression seemed to be contingent upon neighborhood-level contextual factors such as population density and deprivation [47,48]. One article found that relative to married women, widowed women who had increased participation in gardening displayed lower levels of depressive symptoms [49]; another study found that green space was related to less distress for men, but not for women [22]. In addition to self-reported psychological conditions, two studies also included physiological measures of stress. Findings from van Aart et al. [50] suggested significant effects of natural landscapes on self-reported happiness and of industrial landscapes on negative emotions; however, these relationships were not confirmed when using hair cortisol as a measure of stress. Wood et al. [51], using a small sample size of 45, was not able to find significant relationships between childhood nature exposure and either self-reported stress or heart-rate variability (HRV) among adults. 

#### 3.3.3. Reduced Behavioral Problems 

Of the five studies examining children’s behavioral problems, three showed a significant effect of childhood exposure to nature. Although the number of studies was small, neighborhood green space cover, access, use, and quality seemed to serve protective roles against conduct problems in children and adolescents in the majority of the studies [35,40,52]. One study presented partially significant results when exposure and outcome were analyzed cross-sectionally but not longitudinally [50], which only lends support to the notion that concurrent nature exposure has a beneficial effect. Another study found no main effect of outdoor periods in daycare on conduct problems but a significant outdoor duration × age interaction: for children who were offered more outdoor hours, their behavioral problems decreased between the age three and five; but for those allowed fewer outdoor hours, their behavioral problems remained high [53]. 

#### 3.3.4. Increased Cognitive Function 

Studies that assessed either cognitive development in children or decline in older age mostly used an objective test, e.g., the attention network task (ANT) [54], or sets of test batteries that form integrative scales, e.g., the Weschler intelligence scale (WISC) [55]. The findings of these assessments were inconsistent and often depended on the specific measure and age range. For children, greater neighborhood concentration of nature was reported in one article studying a birth cohort as a significant predictor of cognition two years later [56], in another as predicting some cognitive measures, and in yet another as non-predictive of any outcome [39]. For example, Dadvand et al. (2017) examined residential green space since birth and cognitive function at 4–5 years and then at a 7-year follow up. The significant associations observed at 4–5 years of age between residential green spaced (measured by NDVI) and measures of attention (omission errors) disappeared at age 7 when using a different age-appropriate measure [42]. One study used 3D MRIs to examine brain volume and found that clusters (brain regions) associated with residential greenness exposure partly overlapped with clusters related to working memory and inattentiveness [57]. The three studies using childhood nature exposure to predict later-life cognitive decline also showed mixed findings. A study in Chicago determined that public space in good condition was associated with slower rates of cognitive decline [34], but two studies using a particular birth cohort in Scotland found that childhood park availability delayed cognitive decline among older adults only by interacting with adulthood park availability [25] or in areas with low traffic accident rates [58].

#### 3.3.5. Increased Subjective Well-Being 

Of the five studies that examined mental well-being, two reported an advantageous role of earlier nature exposure [36,59]. Ku et al. (2016) confirmed that gardening was associated with greater self-reported well-being in older adults at least 70 years of age. Meanwhile, the other studies tested the relationship between childhood nature visits and well-being in adults and older adults of a wide age range, and demonstrated inconsistent results. Notably, van den Berg and colleagues found a significant association between current nature visit duration and well-being that was modified by childhood nature visits: those who visited nature more in childhood were less likely to benefit from current nature visits [23]. 

### 3.4. Mediators and Moderators

Of the 29 studies included in the review set, six examined potential mediators of the nature–mental health relationship. By including mediators, these studies aim to examine the underlying mechanism or pathway through which nature impacts mental health. Mediators, in other words, answer “how” or “why” questions. The mediators included variables related to personal and parental health, experiences with nature, and other environmental factors (Table 2). The majority of mediators were found to be non-significant. Two of the three studies that examined current nature exposure as a pathway between childhood nature exposure and mental health outcomes reported significant results [37,60], suggesting that more access to nature in childhood may impact an individual’s later-life proenvironmental attitudes or habits of visiting nature, which ultimately impact mental well-being. However, in a British sample, later-life connectedness or perceived satisfaction with nature turned out to be a non-significant pathway [39]. All other mediators were non-significant, except that noise level was significant in a cross-sectional analysis between residential greenness and mental health [50].

Additionally, some studies examined moderators by investigating how personal sociodemographic factors, personal or parental health, experience with nature, and other environmental or contextual factors modified relations between nature and health (Table 2). By including a moderator (i.e., an interaction), these studies considered the extent to which the existence or strength of nature’s effects on health “depends upon” some other (e.g., sociodemographic of contextual) factor. Variables such as childhood nature exposure and parental health have been tested both as mediators and moderators. Overall, the effects of greenness seem to be more pronounced for disadvantaged individuals, including in comparisons of females versus males [25], women who are widowed versus married [49], and those socio-economically disadvantaged versus those advantaged [52]. However, one study displayed diverging evidence that males benefited more from greater early-life nature exposure [22]. Meanwhile, another study revealed a mother’s prenatal BMI to significantly moderate the nature–mental health relationship in children, with stronger effects for children whose mothers had normal rather than high BMI [56]. Three studies investigated the interactions of childhood and current nature exposure, but obtained contradictory results. Two studies by Cherrie and colleagues [25,58] presented a cumulative effect wherein the beneficial effects of childhood greenness on cognition increased when adulthood exposure increased. However, van den Berg et al. [23] showed a more beneficial effect of adulthood greenness on well-being, with lower levels of childhood exposure. Another recurring factor is urbanicity or density. Two studies showed non-significant modification effects of urbanicity; however, other studies demonstrated the effects of nature to be more pronounced in higher-density, anthropogenic land use areas [38,47]. In addition to individual factors, contextual factors were also examined. Childhood nature exposure was more beneficial in deprived neighborhoods [52] and in neighborhoods perceived to be safer [58]. Two studies explored gene-environment interactions in predicting risks of mental health outcomes but found non-significant results [25,45].

### 3.5. Life Stages and Time Span of Nature Exposure

**Figure 5:**
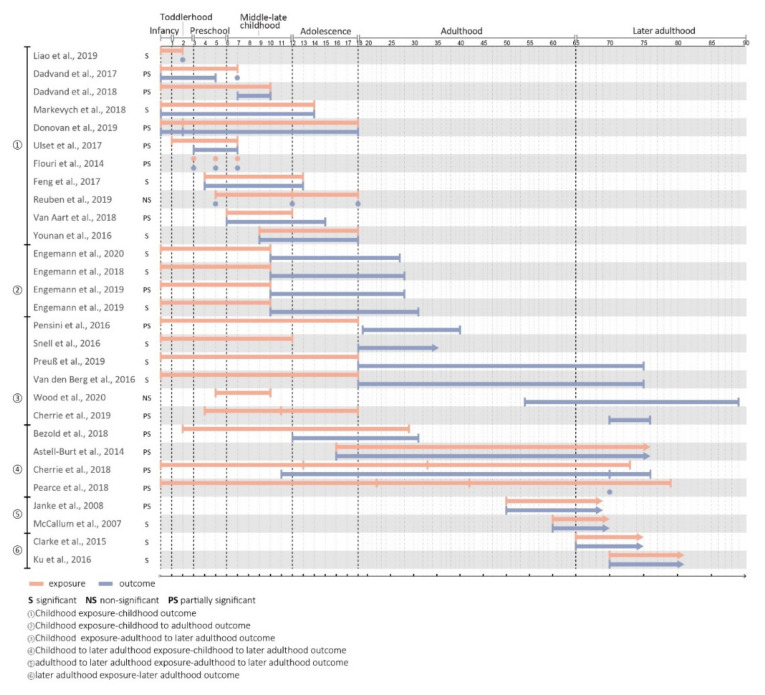
Life stages and ages related to outcomes.

In terms of exposure and outcome across life stages, more interest has been paid to childhood nature exposure than to exposure during adulthood and later adulthood (Figure 5). In sum, 23 articles (79.3%) measured the effects of nature exposure in childhood and adolescence, with the exposure measures starting as early as the birth of the child. Of these, 11 (37.9%) examined mental health outcomes for children and adolescents at various developmental stages, five (17.2%) examined outcomes spanning from childhood to young adulthood, five (17.2%) included outcomes measured in adulthood, and only two (6.8%) involved later adulthood mental health outcomes. Only three studies (10.3%) examined exposure across childhood, adulthood, and later adulthood and mental health outcomes during the same time frame. The remaining four studies (13.8%) focused on exposure and outcomes during the second half of adulthood or later adulthood. Upon synthesizing findings from studies examining the exposures and outcomes at different life stages, there did not seem to be a strong pattern in favor of a critical window beyond which nature exposure stopped having positive effects. In other words, associations between nature experiences and mental health outcomes seemed to be observable across the life span. 

We also cross-examined the life stages with a focus on health outcomes. To date, studies on cognitive development, cognitive decline, or child behavioral problems have focused specifically on the early and late stages of life. Meanwhile, studies examining psychiatric symptoms or mental well-being have used a variety of age ranges and reported somewhat inconsistent results. Due to the small number of studies, it is unclear whether these different findings were attributable to outcomes, measures, populations, or the age range itself. Studies on the incidence of mental disorders have consistently demonstrated a beneficial role of early nature exposure. However, the majority of these studies used childhood nature exposure and adolescent–young adulthood outcomes. Adulthood and later adulthood experiences were underrepresented. 

**Figure 6:**
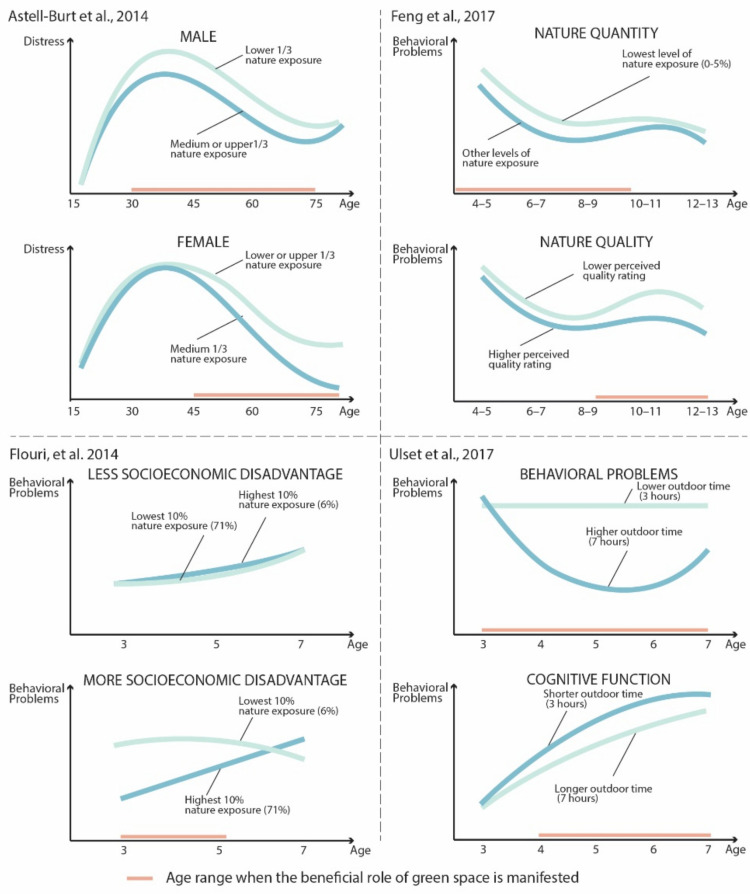
Findings on age ranges at which nature exposure is favorable. (Figure created by the authors for simplicity and comparability based on original figures [22,35,52,[53].)

To explore how the relationship between nature exposure and mental health outcome changes over time, either linearly or non-linearly, several studies included an exposure x age interaction or polynomial term of age. We extracted information from articles that presented plots showing how the relationship between nature exposure and mental health diverged based on age [22,35,52,53], and reproduced the plots to facilitate comparison of the trends (Figure 6). Although these studies examined different populations and outcomes, several observations can be drawn from the commonalities and divergences across the four sets of findings. First, the life trajectory of mental health outcomes over different age ranges seems to be non-linear. Second, in most cases, although higher quantity or quality of nature exposure may pull the curve toward more positive mental health outcomes, it often does not change the overall shape or direction. Lastly, the age ranges when access to nature is most favorable seem to be contingent on gender, SES, measure of exposure, and measure of outcome. For example, Astell-Burt et al. [22] showed that for males, the beneficial effects of green space began to manifest in young adulthood but diminished in older age, while for females, the effects emerged after the mid-40s and remained in older age. Flouri et al. [52] demonstrated that for disadvantaged children between ages 3 and 5, those with less nature exposure have more behavioral problems than those with more exposure; however, the curves converged, even showing signs of reversal at age 7. Finally, Feng et al. [35] documented that a higher amount of natural environment predicted outcomes in children under 10 years old, with the influence of nature quality peaking at 10–11 years. 

## 4. Discussion

### 4.1. Summary of Findings and Implications for Practice

Previous cross-sectional studies support a concurrent association between nature exposure and mental health, and experimental studies have shown significant short-term intervention effects. This systematic review, focusing only on studies that involve early life nature exposure and later life mental health outcomes, demonstrates that although inquiries about the life-long effects of nature have only recently emerged and the number of studies is yet small, the evidence generally suggests a positive effect. Five domains of mental health outcomes have been reported as associated with early-life nature exposure: incidence of mental disorders, psychiatric symptoms and emotions, conduct problems in children, cognitive function, and subjective well-being. The results from studies examining risks of mental disorders as measured by medical or inpatient records show more consistency; however, this may be partly attributable to sample sizes being larger relative to studies reporting other types of outcomes. It may also be that morbidity risks as determined in medical records are more reliable than self-reports. For other mental health outcomes, the observed positive effects are often conditional on the population or measure. In particular, the findings around subjective well-being (SWB) appear to be most inconsistent; it remains questionable whether early-life exposure benefits later-life SWB. Hedonic adaptation theory has long been accepted in SWB research, which states that SWB tends to come back to a personal baseline and the impacts of events such as divorce, unemployment, and death of a loved one can often be eliminated by adaptation over time [61]. It is therefore important to understand whether nature exposure increases SWB and whether this effect can be maintained over time or if it deceases toward the baseline. Similarly, for populations experiencing stressful life events, it is worthwhile to examine whether nature exposure can facilitate a faster return to the baseline level.

Studies included in this review provide evidence for beneficial effects of nature exposure across the life span, although the majority investigated exposure during childhood and adolescence. The outcomes reported also span all life stages. Pooling the findings, there does not seem to be strong evidence that exposure at any given stage is more saliently associated with mental health outcomes, nor that mental health at any particular stage is more affected. However, due to the heterogeneity of the outcome domains, measures, populations, and settings examined, it remains inconclusive whether there is a critical window for nature exposure. From the four longitudinal studies that examined exposure × age interactions, the curve showing mental health outcomes over different age ranges seems to be non-linear and is often conditional on population characteristics. These findings suggest that the association of mental health trajectory with nature exposure is complex and cannot be reduced to a single function that describes all populations. 

Notably, we found limited studies on this topic outside of Europe and North America, and most longitudinal studies were conducted in Europe. More studies in other cultures and geographic areas would help establish the effects of nature across different populations and environmental contexts or definitions of nature. 

The limitations of this study need to be noted. This study adheres to the PRISMA protocols and comprehensive searches were conducted to enhance the rigor of the systematic review. However, meta-bias such as publication bias may still be present [62]. Specifically, studies may be unavailable for inclusion in this review because they found non-significant results and did not achieve publication or because they were published in inaccessible languages. Only peer-reviewed journal articles in English were included in this review, which may incur geographical bias and partially explain the very small number of studies outside of Europe and North America. Furthermore, we conducted the search in July 2020, and more articles have been published on this topic since then [63,64,65]. The results from these studies reported negative associations between early-life nature exposure and later-life anxiety, somatization, and psychiatric disorders, which are in agreement with the results of this review.

Life course studies examining the roles of nature have important policy implications. As promotion of public health shifts attention from the pathogenic model focused on disease and disorder to a salutogenic, holistic approach to health and well-being at all ages, it becomes critical to identify when programs and measures should intervene in individuals’ life spans. Early intervention programs, for example, which set a good foundation for children, may be particularly helpful. For those in disadvantaged neighborhoods or families, policies and programs focused on early life monitoring and intervention may help address inequalities that manifest as severe health disparities in later life. These results may also suggest fresh perspectives for urban planning and environmental policy making. For example, if the effects of nature are age-invariant and linearly cumulative, then promotion of regular, life-long nature exposure would be beneficial. In contrast, the age-variant hypothesis would favor age- or life-stage-graded intervention programs. In such cases, greening efforts targeting specific ages, such as in early childcare centers, schools, and neighborhood playgrounds, would provide more benefits than increasing urban canopies evenly in cities. With more conclusive findings, environmental design and planning policies can develop age-appropriate environments that best leverage the benefits of nature [66]. 

### 4.2. Insights from Qualitative Studies on Life Course Nature Exposure and Mental Health

During our review process, we identified a total of nine qualitative studies evaluating the connection between past experiences in nature and mental well-being. These used approaches such as semi-structured interviews, geo-narratives, or photovoice techniques (Supplementary Material III). Although these studies were excluded from our review protocol, we considered them separately to form comparisons and yield additional insights. 

The qualitative studies employed broader definitions of “nature exposure”, including rarely-discussed types of environments and exposure levels. For example, Bell and colleagues [67,68] highlighted the therapeutic value of blue spaces such as coastal environments. The activity types in nature that these studies captured ranged from quiet or passive recreation to physical activity to extreme sports [69]. The concept of certain natural environments (e.g., woodland) being “risky environments” was also explored in depth, with young adults in northwestern England ascertaining their perceived benefits from childhood play in nearby woods (e.g., increased confidence in assessing risk, heightened inner strength, and sense of agency), however also expressing concerns such as parental anxiety and fear of being trapped [70]. 

Consistent with our findings, qualitative studies have also supported the mental health benefits of nature exposure across childhood [71], adolescence [72], adulthood [73], and later adulthood [74]. In particular, both quantitative and qualitative studies have consistently highlighted formative childhood experiences related to nature; for example, the majority of adult interviewees in Winnipeg, Canada described formative childhood experiences related to gardening [71]. However, the qualitative studies suggested multi-pathway effects and interactions between activity–development and formal–informal processes, showing the complexities that were often not revealed in quantitative studies. The role of nature in coping with aging was also discussed using qualitative methods. For older women in rural areas, positive childhood experiences with nature set a trajectory of strong emotional attachment to and interactions with nature. In addition, participants’ attention and attachment to nature became more important during middle and later adulthood [75]. For older adults transitioning into later life and dementia care, nature provided grounding in past social and cultural identity, promoted healthy behaviors, and contributed to feelings of connection as a place-based anchor [74]. 

Qualitative studies placed more emphasis on populations that go through life transitions and on the underlying perception and attitude changes that drive behavioral and mental health outcomes related to nature. Different natural environments afforded varied experiences and were used differently at different life stages depending on the therapeutic needs of the individual. For example, as participants underwent major life transitions such as becoming a parent or at the onset of illness or impairment, the specific blue and green spaces they frequented changed with their well-being priorities and needs [67,68]. Similarly, the social dimensions of nature interaction also shifted across life stages. Girls in early adolescence used local parks as settings for social interactions, but upon entering middle and late adolescence, they began to use them as escape retreats [72]. Perceptions of nature also often change across the lifespan, with Husser [75] finding that older women’s appreciation and reliance on nature as a coping resource grew as they aged. 

While qualitative studies do not statistically examine the relations among nature and health variables, they do provide rich and valuable insights that can be further examined in subsequent studies. Qualitative research suggests that additional studies might focus on the effects of “everyday nature” and a variety of wild to manicured settings and exposure, effect modification or complex pathways through personal activities and emotional attachment, and effects during life transitions and events. 

### 4.3. Validity Concerns and Methodological Considerations

In this section, we highlight salient issues in the reviewed literature related to each of the four core types of validity, namely internal, external, construct, and statistical validity, as well as potential strategies and methods to address these challenges. 

#### 4.3.1. Internal Validity

Although the majority of studies on nature and mental health have been cross-sectional, many articles identified in this review are longitudinal. As random controlled trials or even quasi-experiments are challenging for interventions related to nature exposure, which can happen anywhere and anytime over a lifetime, cross-sectional studies with carefully designed inclusion–exclusion criteria and statistical control of confounding variables can improve internal validity. Compared to cross-sectional studies, longitudinal and especially cohort studies offer stronger evidence toward establishing causal relationships. Additionally, studies using cohort designs or linked longitudinal health data enable movement in the direction of testing a wide range of mental health disorders (e.g., schizophrenia, bipolar) that have not been discussed before. However, even longitudinal studies that examine life course trajectories are often not adequate to establish mechanistic pathways, making it difficult to determine whether environmental exposure is cause or correlate of mental health outcomes. These studies are often impacted by maturation effects and a myriad of other personal, social, and environmental factors that occur in the long term—particularly when the cohort is not selected based on exposure to nature, which is the case for almost all cohort studies reviewed. In addition, different instruments are sometimes needed as children develop cognitively and emotionally, and best practices in data collection change over time, which also presents threats to establishing causality.

#### 4.3.2. External Validity

Another challenge in nature and health research is generalizability. We must often ask: do these findings apply to another setting? To another population? To another age group? To another culture? With time and the continued explosion of research on this topic, these questions may be addressed. Perhaps more challenging is generalizing and translating findings into public health and urban planning policy and practice. Life course approaches inherently emphasize effects over the long term; as a result, external validity is critically important. A unique challenge of life course studies is how to maintain representativeness of the population over a long period of time. As inter-wave periods tend to be longer than in typical longitudinal studies, a larger proportion of the sample may drop out. In addition, birth, death, migration, urbanization, or suburbanization may cause the population composition to change, rendering it difficult to track and maintain a representative sample. Another important limitation is that many of the reviewed studies used a subset of an existing cross-sectional or longitudinal dataset, which had a sample frame designed for a different set of research questions; accordingly, the representativeness of the study population can be hard to evaluate. Furthermore, attrition rates are difficult to calculate when using a subset of an existing dataset. As such, more discussion related to external validity when using sub-samples from existing datasets is warranted. Study designs and sampling frames developed specifically to evaluate the effects of nature exposure on health would offer more insights, such as the Positive Health Effects of the Natural Outdoor Environment in Typical Populations in Different Regions in Europe (PHENOTYPE) project. As Scandinavian countries and countries in other regions develop national register linkages to merge health-related datasets, including mortality, morbidity, and even bio- and genetic samples, opportunities will emerge for personal history tracing and relating environmental exposure to health outcomes.

#### 4.3.3. Construct Validity

The studies included in this review used a variety of outcome measures, ranging from validated self-report instruments and medical records to objective cognitive test batteries and physiological measures such as HRV and neuroimaging. Most are validated measurements of mental health outcomes. However, the issue of how to measure the construct life course or long-term exposure to nature remains a question untouched in the literature. Therefore, we discuss the challenges related to measuring lifelong exposure to nature and new technologies and possibilities.

The literature has classified nature exposure into three levels: access, exposure, and engagement [76]. In our review set, the cross-sectional studies mostly employed retrospective, self-reported measures of childhood nature exposure or visits. This method aims to capture frequencies or durations of an individual’s nature visits, but is subject to construct validity threats such as self-report bias, mono-operation bias (i.e., if just one item is used), and information bias (e.g., when there is recall error). For example, when retrospectively determining childhood nature exposure in adulthood or later adulthood, the recall period is fairly long, and health conditions of participants, along with the fallibility of memory, may impact their ability to accurately report past exposure [77]. To our knowledge, no study has evaluated the magnitude and distribution of the recall error related to such nature exposure measures. A second method for retrospectively assessing nature exposure is to ask for the residential histories (addresses) of participants and then analyze the surroundings using environmental datasets. One example is the life grid approach, which was employed in only a few reviewed studies [25] but is used widely in other topics within life course epidemiology. These questionnaires use a grid layout to collect information related to life events, residences, and other variables [66], which is well-suited for gathering information about both residential locations and the impactful life events and turning points that are critical to life course research. 

Most longitudinal studies used objective availability or accessibility measures of green space—mostly NDVI and land cover characteristics surrounding participants’ residential addresses. When the same exposure measures are available for each wave of data collection, this approach could capture lifelong changes in individuals’ exposures and examine the growth curve of the relationship. However, difficulty arises when environmental data are only available for recent years, or on a timeframe that does not match the outcome measures. These challenges present an “instrumentation” threat to construct validity, i.e., the measure or operationalization of the construct changes over time. To address this challenge, some studies have used georeferenced older maps to identify historical green space availability or included only the waves or participants with available data [48]. Going forward, the recent growth of satellite and aerial imagery and LiDAR technologies provides huge potential for extracting high-resolution measures of green space and for capturing three-dimensional characteristics of vegetation.

Only a few studies have used multiple measures to operationalize nature as the independent variable [35,36]; those that do not are vulnerable to mono-method bias, which is a threat to construct validity. Ideally, researchers would combine multiple measures such as objective quantification of nature, participants’ self-reports, and reports of others (such as parents or siblings) to both guard against the threat of mono-method bias and to cross-validate measures against one another by examining convergent validity. The development of tracking technologies, activity space delineations, and ecological momentary assessment and geographic ecological momentary assessment should greatly assist in quantifying individuals’ exposure to and assessments of natural features in their daily environments and should capture the engagement aspect (e.g., active or passive activity, amount of attention paid to nature, and informal or formal educational) that is important for health benefits [78,79]. Many studies consider greenness or nature exposure as a broad construct. However, more detailed considerations, such as the type of vegetation, plant species, biodiversity, and location or placement factors, may also be important to capture [80,81]. 

#### 4.3.4. Statistical Conclusion Validity

As epidemiological studies of nature and mental health have become increasingly common, sample size presents a salient threat to statistical validity, as these studies routinely employ large sample sizes. Very large samples can make analyses vulnerable to a type 1 error or false alarm. Similarly, if analyses are conducted at the neighborhood, regional, or national level, the ecological fallacy may threaten statistical validity if conclusions are made at the individual level. 

Cross-sectional studies included in this review employed regression models, partial correlation analyses, or multilevel models (MLMs). Longitudinal studies mostly employed MLMs and Cox regression, with a few using generalized estimating equations (GEEs) or (latent) growth curve models (LGCs). Since longitudinal studies need to consider that repeated measures are nested within individuals, simple regression analyses are often inadequate. Multilevel models account for such nesting and allow variances for the intercept or slope, thus accounting for changes in baseline and development rates over time or measurements. They can also incorporate additional levels, such as the neighborhood or district level, which can potentially address ecological fallacies. Cox regression models and other survival models consider the time-to-event aspect of the development of certain morbidity and mortality outcomes, and are therefore suitable for investigating early nature exposure and the onset of mental health disorders. However, these are also subject to censoring effects and are less useful for outcomes such as subjective well-being. GEEs provide another robust method of estimating models for longitudinal and clustered data. Latent growth curve models were used only in a few of the reviewed articles, but have gained popularity in life course epidemiology [82] due to their flexibility in fitting any functional form that represents the trajectory [83], especially latent growth mixture modeling or latent class growth modeling. Finally, as moderation or mediation analyses are often needed in this kind of research, the structural equation modeling (SEM) framework is promising; it can incorporate multi-level, latent, and mediation or moderation pathways simultaneously.

Most studies either used complete case analysis or did not report how missing data was handled. As the life course approach often requires multiple waves of cohort data or the linkage of a variety of data, missing data can be an important issue. Often it may not be appropriate to assume data are missing completely at random, and imputation methods may be appropriate. Sensitivity analysis may also be helpful in this area. 

### 4.4. Unanswered Questions and Future Directions within the Life Course Perspective

In addition to discussing a number of methodological issues, this review has also identified gaps in knowledge and opportunities for future research, especially with respect to the life course approach to the study of nature and mental health. These unanswered questions can guide theoretical development and refocus future research.

#### 4.4.1. Timing in Lives

A core principle of the life course approach is timing in lives; in other words, the phase of life at which an event occurs in an individual’s life matters. The critical period model of the life course framework [17] claims that exposure during a certain developmental stage alters physical, psychological, or cognitive functioning, resulting in permanent health outcomes. Previous studies point to an individual’s connection to nature having life-stage dependent effects [84]. However, the evidence we reviewed on mental health outcomes did not reveal a clear critical period for acquiring the benefits of nature; rather, the benefits seem to manifest across life stages. As childhood and older adulthood are often important stages when mental health changes occur, these stages have received more research attention; however, conclusive evidence is yet lacking regarding whether nature exposure during specific periods is more critical.

- Are the mental health benefits of nature gained at all life stages or do they depend on stage?
- Is there a critical window when lack of nature exposure may cause irreversible results? Is exposure during childhood or later adulthood more important for lifelong mental health than at other stages?
- If there is a critical period for establishing behavioral or biological advantages from nature exposure, are the effects modifiable by exposure later in life?

#### 4.4.2. Life Course Trajectories, Transitions, and Turning Points

A related notion that is central within the life course perspective is the potency of early life experience in terms of setting an individual on a life course trajectory. A trajectory is a stable pattern of behavior across the life course that sets an individual on path toward a likely outcome. Trajectories are “sticky”; in other words, they have inertia, which makes them powerful, self-reinforcing, and difficult to change [85]. Early experiences may place individuals onto a life trajectory that may eventually impact health outcomes [17,29,86]. The literature has shown links between childhood nature exposure and environmentalism [16,87], which may be related to later-life mental health. 

Consistent with the notion of trajectory, the cumulative model of life course theory [17] states that benefits or risks at earlier stages accumulate to influence risks during adulthood. Thus, nature exposure across different life stages may build up as “nature capital”, which offers immunization effects, bolstering resilience. This is especially relevant when discussed within the life transitions context, when a change in responsibilities or social roles occurs, such as becoming a parent, starting a new job, or retirement [85,88]. In some cases, a transition can also be a turning point at which an individual breaks from a current trajectory and shifts to a new trajectory. The role of prior or concurrent nature exposure during these life transitions or turning points has not been adequately studied.

- Are the protective benefits of nature cumulative?
- How do life course transitions such as becoming a parent, the death of a loved one, or retiring from one’s job relate to time spent in nature? How does this relate to subsequent mental health outcomes?
- Are some life course transitions more likely than others to become turning points, such that people shift toward greater engagement with nature and to a more positive mental health life course trajectory?

#### 4.4.3. Cultural and Contextual Influences

Cultural and contextual influences are external events and cultural phenomena that affect developmental or health outcomes [89,90]. These include neighborhood influences such as poverty and deprivation, as well as macro historical events such as economic depression, wars, and pandemics. The literature has demonstrated that early life stress profoundly impacts future mental health trajectories. For example, both current and past neighborhood SES have lagged effects on mental health [91]. Other evidence suggests that nature may buffer (i.e., dampen) the effects of adverse factors such as poverty, stress, or financial strain on mental health [92,93], or may enhance the capacity to cope with poverty [94,95]. External stressors such as the COVID-19 pandemic may also have profound mental health impacts [96,97]. Little is known, however, about how access to nature might interact with the timing and sequencing of contextual stress and adversity over the life course.

- Does nearby nature, as a neighborhood contextual factor, moderate the stress–mental health relationship over time? How might this be relevant in the context of the global COVID-19 pandemic?
- Are there important pathways through which early contextual factors influence later outcomes?

#### 4.4.4. Linked Lives

Linked lives, within the life course perspective, examines how interdependency between individuals influences development. This can include parent–child dyads, spouses, or other pairings that mutually affect behavior over time [98]. Several studies examined the prenatal or at-birth environment and children’s health outcomes [41,56], while the parent-child linkages remain to be elucidated. Autobiographical reminiscence studies of significant life events point to the role of an influential individual (parent, scout leader, grandparent, etc.) along with spending time in nature during youth as factors that contribute to later life connection to nature and environmental commitment [77]. Regarding nature–mental health research, future studies might similarly examine the role of mother–child connections to nature, or spousal partnerships.

- What is the role of family members in connecting a child to nature and, ultimately mental health outcomes?
- How might an individual’s nature connection influence their spouse’s time in and connection to nature as well as their navigation of life course transitions and subsequent mental health outcomes?

## 5. Conclusions

Overall, the results of this systematic review suggest a beneficial role of early nature exposure in later-life mental health. While inconsistencies and methodological challenges exist, these recent studies around the life course benefits of nature exposure provided new theoretical insights and policy implications. Future studies should pay attention to the validity challenges outlined. More investigations are needed to fill the knowledge gaps related to the trajectories and mechanisms underlying the associations between life course nature exposure and mental health. 

## Figures and Tables

**Table 1:** Characteristics of included studies.

	Article	Geography		Population		Study Design		Nature Exposure			Mental Health Outcome		Confounders
		Country	Urbanicity	Population ^1^	Sample Size ^2^	Type of Study	Dataset	Setting	Type	Measurement	Domain	Measurement	
1	Astell-Burt et al., 2014	UK	Urban	Adults (16+ year)	2681 wards; 65,407 person-years	Longitudinal	British Household Panel Survey (BHPS)	Neighborhood greenness	Availability/density/cover	Land cover class (%; green and natural environment)	Psychiatric symptoms/psychological conditions	General Health Questionnaire (GHQ)	Demographics (e.g., age) SES (e.g., household income) Health behavior self/family (e.g., smoking status)
2	Bezold et al., 2018	US	NR	Children and early adolescents (9–14 year)	8374 persons	Longitudinal	Growing Up Today Study (GUTS)	Neighborhood greenness	Availability/density/cover	NDVI	Psychiatric symptoms/psychological conditions	McKnight Risk Factor Survey (MRFS) The Center for Epidemiologic Studies ten-item depression scale (CES-D 10)	Demographics (e.g., age) SES (e.g., household income) Health record self/family (e.g., maternal history of depression)
3	Cherrie et al., 2018	UK	Mixed	Adults born in 1936	281 persons	Longitudinal	Lothian Birth Cohort 1936	Neighborhood greenness	Availability/density/cover	Public park availability (%)	Cognitive function	Moray House Test No.12 (MHT)	Demographics (e.g., sex) SES (e.g., occupation) Health behavior self/family (e.g., childhood/adulthood smoking)
4	Cherrie et al., 2019	UK	NR	Adults born in 1936	281 persons	Longitudinal	Lothian Birth Cohort 1936	Neighborhood greenness	Availability/density/cover	Public park availability (%)	Cognitive function	Moray House Test No.12 (MHT)	Demographics (e.g., sex) SES (e.g., occupation) Health behavior self/family (e.g., childhood/adulthood smoking)
5	Clarke et al., 2015	US	Urban	Older adults (65+ year)	82 block groups; 6158 persons	Longitudinal	Chicago Health and Aging Project (1993–2011)	Neighborhood greenness	Quality	Quality of public space audit	Cognitive function	East Boston Memory Test (EBMT) Symbol digit modalities test (SDMT) Mini Mental State Examination (MMSE)	Demographics SES Health record self/family (e.g., chronic conditions) Social network (e.g., size) Environment (e.g., sidewalk)
6	Dadvand et al., 2017	Spain	Urban	Pregnant women (16+ year)	888/987 persons	longitudinal	INfancia y Medio Ambiente (INMA)	Neighborhood greenness	Availability/density/cover	NDVI Residential surrounding tree cover (VCF)	Cognitive function	Conners’ Kiddie Continuous Performance Test (K-CPT) Attentional Network Task (ANT)	Demographics SES (e.g., maternal education) Health record self/family (e.g., maternal smoking) Environment (e.g., neighborhood SES)
7	Dadvand et al., 2018	Spain	Urban	Children (7–10 year)	39 schools; 253/2897 persons	Longitudinal	Brain Development and Air Pollution Ultrafine Particles in School Children (BREATHE)	Neighborhood greenness	Availability/density/cover	NDVI	Cognitive function Brain development	N-back Working Memory Test (WT) Attentional Network Task (ANT) Brain region peak voxel gray/white matter	Demographics (e.g., sex) SES (e.g., maternal education)
8	Donovan et al., 2019	New Zealand	Mixed	Children born in 1998	49,923 persons;	Longitudinal	New Zealand’s Integrated Data Infrastructure (IDI)	Neighborhood greenness	Availability/density/cover	NDVI Land cover class (%) Urbanicity (urban vs. rural)	Incidence of mental disorders	Reported incidence	Demographics (e.g., sex) SES (e.g., parental education) Health record self/family (e.g., infections) Environment (e.g., traffic-related air pollution)
9	Engemann et al., 2018	Denmark	Mixed	Adults born 1985–2003	943,027 persons	Longitudinal	Danish Civil Registration System, Danish Psychiatric Central Research Register	Neighborhood greenness	Availability/density/cover	NDVI	Incidence of mental disorders	Reported incidence	Demographics (e.g., sex) SES (e.g., parental education)
10	Engemann et al., 2019 a	Denmark	Mixed	Adults born 1985–2003	943,027 persons	Longitudinal	Danish Civil Registration System, Danish Psychiatric Central Research Register	Neighborhood greenness	Availability/density/cover	NDVI	Incidence of mental disorders	Reported incidence	Demographics (e.g., sex) SES (e.g., parental education) Health record self/family (e.g., parents’ records of psychiatric disorder) Environment (e.g., neighborhood SES)
11	Engemann et al., 2019 b	Denmark-Europe	Mixed	Adults born 1985–2003	943,027 persons	Longitudinal	Danish Civil Registration System, Danish Psychiatric Central Research Register	Neighborhood greenness	Availability/density/cover	Land cover class (%; urban, agriculture, near-natural green space, and blue space) NDVI	Incidence of mental disorders	Reported incidence	Demographics (e.g., sex) SES (e.g., parental education) Health record self/family (e.g., parents’ records of mental illness) Environment (e.g., neighborhood SES)
12	Engemann et al., 2020	Denmark-Europe	Mixed	Adults born 1981–2005	19,746 persons	Longitudinal	iPSYCH2012 case-cohort sample	Neighborhood greenness	Availability/density/cover	NDVI	Incidence of mental disorders	Reported incidence	Demographics (e.g., sex) SES (e.g., parental education)
13	Feng et al., 2017	Australia	Mixed	Children (4–5 year)	4968 persons	Longitudinal	Longitudinal Study of Australian Children (LSAC)	Neighborhood greenness	Mixed (Availability/density/cover, Quality)	Land use class (%; parkland) Self-reported quality	Emotional/conduct problems	Strengths and Difficulties Questionnaire (SDQ)	Demographics (e.g., sex) Environment (e.g., neighborhood SES)
14	Flouri et al., 2014	UK	Urban	Children born 2000–2001	6384 persons	Longitudinal	Millennium Cohort Study	Neighborhood greenness	Mixed (Availability/density/cover, Frequency/duration)	Land use class (%; green space domestic gardens, fresh water) Sole access to a garden Frequency of park visit	Emotional/conduct problems	Strengths and Difficulties Questionnaire (SDQ)	Demographics (e.g., sex) SES Health record self/family (e.g., maternal psychological distress) Health behavior self/family (e.g., physical activity) Environment (e.g., neighborhood deprivation) Adverse life events
15	Janke et al., 2008	US	NR	Women who became widowed 1986–1989 or 1989–1994	296 persons	Longitudinal	Americans Changing Lives (ACL)	Gardening as leisure activity	Frequency/duration	Frequency of informal, formal, and physical leisure activities, including gardening	Psychiatric symptoms/psychological conditions	The Center for Epidemiologic Studies ten-item depression scale (CES-D 10)	Demographics SES
16	Ku et al., 2016	Taiwan	NR	Older adults (70+ year)	1268	Longitudinal	Survey of Health and Living Status of the Elderly	Gardening as leisure activity	Frequency/duration	Frequency of engagement in leisure activity, including gardening	Mental well-being	Life Satisfaction Index A (LSIA)	Demographics (e.g., sex) SES (e.g., educational level) Health record self/family (e.g., depressive symptoms) Health behavior self/family (e.g., physical activity)
17	Liao et al., 2019	China	Mixed	Women who became pregnant 2012–2015	1312 mother–child pairs	Longitudinal	NA	Neighborhood greenness	Availability/density/cover	NDVI	Cognitive function	Bayley Scales of Infant Development (BSID)	Health behavior self/family (e.g., physical activity) Environment (e.g., PM2.5)
18	Markevych et al., 2018	Germany	Mixed	Children born 2000–2004	186 postal code areas; 66,823 persons	Longitudinal	AOK PLUS statutory health insurance company dataset	Neighborhood greenness	Availability/density/cover	NDVI	Incidence of mental disorders	Reported incidence	Demographics (e.g., sex) SES (e.g., unemployment) Environment (e.g., air pollution)
19	McCallum et al., 2007	Australia	Semi-urban	Older Adults (60+ year)	2805 persons	Longitudinal	Dubbo Study of the Elderly	gardening as leisure activity	Frequency/duration	Frequency of gardening	Incidence of mental disorders	Reported incidence	Demographics (e.g., age) SES (e.g., education) Health record self/family (e.g., stroke) Health behavior self/family (e.g., walking) Environment (e.g., neighborhood SES)
20	Pearce et al., 2018	UK	NR	Adults born in 1936	23 wards; 328/531/1091 persons	Longitudinal	Lothian Birth Cohort 1936	Neighborhood greenness	Availability/density/cover	Public park availability (%)	Psychiatric symptoms/psychological conditions	Hospital Anxiety and Depression Scale (HADS)	Demographics (e.g., age) SES (e.g., education) Health behavior self/family (e.g., smoking)
21	Pensini et al., 2016 ^3^	Australia	NR	Adults (19–40 year)	646 persons	Cross-sectional	NA	Childhood nature experience	Frequency/duration	Frequency of time spent in 13 types of natural environments (Natural Environment Exposure Scales [NEES])	Mental well-being	Warwick-Edinburgh Mental Well-Being Scale (WEMBS) Ryff Scales of Psychological Well-Being (PWB) Meaning in Life Questionnaire (MLQ)	Current nature exposure
		Germany			141 persons								
22	Preuß et al., 2019	Spain, the Netherlands, Lithuania, and UK	Urban	Adults (18–75)	30 neighborhoods; 3585 persons	Cross-sectional	Positive Health Effects of the Natural Outdoor Environment in Typical Populations in Different Regions in Europe (PHENOTYPE)	Childhood nature experience	Frequency/duration	Frequency of visits to natural outdoor environment (NOE) during childhood	Mental well-being	Short-Form Health Survey (SF-36)	Demographics (e.g., age) SES (e.g., education) Health behavior self/family (e.g., smoking) Environment (e.g., neighborhood SES)
23	Reuben et al., 2019	UK	Urban and suburban	Twins born 1994–1995	1658 persons	Longitudinal	Environmental Risk (E-Risk) Longitudinal Twin Study	Neighborhood greenness	Availability/density/cover	NDVI	Cognitive function	Weschler Intelligence Scale (WISC) Cambridge Neuropsychological Test Automated Battery (CANTAB)	Demographics (e.g., age) SES (e.g., education) Environment (e.g., neighborhood SES)
24	Snell et al., 2016	Australia	NR	Adults (18+ year)	300 persons	Cross-sectional	NA	Childhood nature experience	Frequency/duration	Frequency of visits to four different natural environments during childhood	Psychiatric symptoms/psychological conditions	Depression Anxiety and Stress Scales (DASS)	Current nature exposure
25	Ulset et al., 2017	Norway	Suburban	Children (1–6 year)	28 daycare centers; 562 individuals; 2136 person-years	Longitudinal	NA	School greenness	Frequency/duration	Duration of time outside at daycare centers of two different types (nature-based vs. conventional)	Emotional/conduct problems Cognitive function	Strengths and Difficulties Questionnaire (SDQ) Weschler Intelligence Scale (WISC)	Demographics (e.g., age) SES (e.g., education) Health record self/family (e.g., parental inattention-hyperactivity) Environment (e.g., daycare center quality)
26	van Aart et al., 2018	Belgium	Semi-urban	Children (around 7–12 year)	172/224 persons	Longitudinal	Identification and prevention of dietary- and lifestyle-induced health effects in children and infants project (IDEFICS)	Neighborhood greenness	Availability/density/cover	Land cover class (%; semi-natural, forested, agriculture, industrial, residential)	Emotional/conduct problems Psychiatric symptoms/psychological conditions	Recent feelings of happiness, sadness, anger, and anxiousness Strengths and Difficulties Questionnaire (SDQ) Hair cortisol	Demographics (e.g., age) SES (e.g., parental education) Environment (e.g., air pollution)
27	van den Berg et al., 2016	Spain, the Netherlands, Lithuania, and UK	Urban	Adults (18–75 year)	30 neighborhoods; 3748 persons	Cross-sectional	PHENOTYPE	Childhood nature experience	Frequency/duration	Frequency of time spent in natural environments during childhood	Mental well-being	Short-Form Health Survey (SF-36)	Demographics (e.g., age) SES (e.g., education)
28	Wood et al., 2020	UK–Europe	NR	Adults (54–89)	45 persons	Cross-sectional	NA	Childhood nature experience	Frequency/duration	Frequency of childhood nature exposure	Mental well-being Psychiatric symptoms/psychological conditions	Warwick-Edinburgh Mental Well-Being Scale (WEMBS) Perceived Stress Scale (PSS) Heart Rate Variability (HRV)	
29	Younan et al., 2016	US	Urban	Twins and triplets born 1990–1995	640 families; 1287 persons	Longitudinal	Risk Factors for Antisocial Behavior (RFAB) twin study	Neighborhood greenness	Availability/density/cover	NDVI	Emotional/conduct problems	Child Behavior Checklist (CBCL)	SES Environment (e.g., neighborhood quality)

^1^ Age group at the onset of the study. ^2^ In the case of multilevel analysis (e.g., cohort/neighborhood, person, person-year), all levels of reported sample sizes reported in the article were included.

**Table 2:** Mediators and moderators investigated in reviewed studies.

Domain	Mediators/Pathways			Moderators/Effect Modifiers		
	Variables	No. of Studies Testing the Mediator	No. of Studies Finding Significant Mediation	Variables	No. of Studies Testing the Moderator	No. of Studies Finding Significant Moderation
Sociodemographic				Age/Age^2^/Age^3^	5	5/5
				Gender	2	2/2
				Marital status	1	1/1
				Socioeconomic status	2	2/2
Personal/parental health and physical activity	Parents’ mental and physical health	2	0/2	Parents’ mental and physical health (BMI)	1	1/1
	Parents’ physical activity	1	0/1			
	Physical health	1	0/1			
	Physical activity	1	0/1			
Experience with nature	Current/adulthood nature exposure	3	2/3	Current/adulthood nature exposure	3	3/3
	Perceived amount of nature available	1	0/1			
	Connectedness with nature	1	0/1			
Environmental and contextual	PM2.5	2	0/2			
	Noise	1	1/1			
				Density	1	1/1
				Land use	1	1/1
				Urbanicity/urbanization	2	0/2
				Perceived safety (traffic accidents)	1	1/1
				Neighborhood deprivation	1	1/1
Others				Genetics	2	0/2
				Adverse life events	1	0/1

## Data Availability

No new data were created in this study other than those included in Table 1 and Supplementary Materials.

## Supplementary Materials

The following are available online at https://www.mdpi.com/article/10.3390/ijerph18105146/s1: Supplementary Material I. Search Syntax for Web of Science. Supplementary Material II. Table S1. Study Quality Assessment Results. Supplementary Material III. Table S2. Qualitative Studies Identified and Reviewed.
