# Supplementary material for: Life Course Nature Exposure and Mental Health Outcomes: A Systematic Review and Future Directions
Source: Int J Environ Res Public Health. 2021 May 12;18(10):5146. doi: 10.3390/ijerph18105146 (PMC8152056; doi:10.3390/ijerph18105146)
Supplement: Supplementary file 1 [file ijerph-18-05146-s001.zip › ijerph-1171051-supplementary.pdf]

International Journal of
Environmental Research and Public Health

MDPI

Supplementary Material I.

Search Syntax for Web of Science

TS = ((green-space* or green-and-blue-space* or green-or-blue-space* or greenspace* or green-place* or greenery or greenness or "nearby nature" or "access to nature" or "exposure to nature" or "urban nature" or "connectedness to nature" or "connection to nature" or natural-environment* or natural-setting* or park or parks or tree-canop* or "tree cover" or garden* or urban-forest* or vegetation or "outdoor recreation" or "outdoor play" or ecotouris* or wilderness)

AND

(life-course or life-stage* or life-trajector* or childhood or adolescence or adulthood or life-path* or life-transition* or life-chang* or lifelong or life-long or grow-up or growing-up or grown-up* or grownup* or early-life or later-life or later-in-life or life-experience* or turning-point* or reminiscen*))

Supplementary Material II.

Table S1. Study Quality Assessment Results.

	Study	Assessment Item*														Total	Quality rating
		**1**	**2**	**3**	**4**	**5**	**6**	**7**	**8**	**9**	**10**	**11**	12	13	14		
1	Astell-Burt et al., 2014	Y	Y	NA/NR	Y	N	N	Y	Y	Y	N	Y	NA/NR	NA/NR	Y	8	Fair
2	Bezold et al., 2018	Y	Y	NA/NR	Y	N	Y	Y	Y	Y	Y	Y	NA/NR	NA/NR	Y	10	Good
3	Cherrie et al., 2018	Y	Y	NA/NR	Y	N	Y	Y	Y	Y	Y	Y	NA/NR	Y	Y	11	Good
4	Cherrie et al., 2019	Y	Y	NA/NR	Y	N	Y	Y	Y	Y	Y	Y	NA/NR	NA/NR	Y	10	Good
5	Clarke et al., 2015	Y	Y	Y	Y	N	N	Y	Y	Y	N	Y	NA/NR	Y	Y	10	Good
6	Dad-vand et al., 2017	Y	Y	NA/NR	Y	N	Y	Y	Y	Y	Y	Y	NA/NR	N	Y	10	Good
7	Dad-vand et al., 2018	Y	Y	NA/NR	Y	N	Y	Y	Y	Y	N	Y	NA/NR	NA/NR	Y	9	Good

8	Donovan et al., 2019	Y	Y	NA/NR	Y	N	Y	Y	Y	Y	Y	Y	NA/NR	Y	Y	11	Good
9	Engemann et al., 2018	Y	Y	NA/NR	Y	N	Y	Y	Y	Y	Y	Y	NA/NR	Y	Y	11	Good
10	Engemann et al., 2019b	Y	Y	NA/NR	Y	N	Y	Y	Y	Y	Y	Y	NA/NR	Y	Y	11	Good
11	Engemann et al., 2019a	Y	Y	Y	Y	N	Y	Y	Y	Y	Y	Y	NA/NR	Y	Y	12	Good
12	Engemann et al., 2020	Y	Y	Y	Y	N	Y	Y	Y	Y	Y	Y	NA/NR	NA/NR	Y	11	Good
13	Feng et al., 2017	Y	Y	Y	Y	N	N	Y	Y	Y	N	Y	NA/NR	N	Y	9	Good
14	Flouri et al., 2014	Y	Y	NA/NR	Y	N	Y	Y	Y	Y	N	Y	NA/NR	NA/NR	Y	9	Good
15	Janke et al., 2008	Y	Y	NA/NR	Y	N	Y	Y	Y	Y	Y	Y	NA/NR	NA/NR	Y	10	Good
16	Ku et al., 2016	Y	Y	NA/NR	Y	N	Y	Y	Y	Y	Y	Y	NA/NR	Y	Y	11	Good
17	Liao et al., 2019	Y	Y	Y	Y	N	Y	Y	Y	Y	N	Y	NA/NR	Y	Y	11	Good

18	Markevych et al., 2018	Y	Y	NA/NR	Y	N	Y	Y	Y	Y	Y	Y	NA/NR	NA/NR	Y	10	Good
19	McCallum et al., 2007	Y	Y	Y	Y	N	Y	Y	N	Y	N	Y	NA/NR	NA/NR	Y	9	Good
20	Pearce et al., 2018	Y	Y	NA/NR	Y	N	Y	Y	Y	Y	Y	Y	NA/NR	NA/NR	Y	10	Good
21	Pensini et al., 2016	Y	Y	NA/NR	N	N	N	Y	Y	Y	N	Y	NA/NR	NA/NR	N	6	Fair
22	Preuß et al., 2019	Y	Y	NA/NR	Y	N	N	Y	Y	Y	N	Y	NA/NR	NA/NR	Y	8	Fair
23	Reuben et al., 2019	Y	Y	Y	Y	N	Y	Y	Y	Y	Y	Y	NA/NR	Y	Y	12	Good
24	Snell et al., 2016	Y	Y	NA/NR	N	N	N	Y	Y	Y	N	Y	NA/NR	NA/NR	N	6	Fair
25	Ulset et al., 2017	Y	Y	Y	Y	N	N	Y	Y	Y	N	Y	NA/NR	Y	Y	10	Good
26	Van Aart et al., 2018	Y	Y	NA/NR	Y	N	Y	Y	Y	Y	N	Y	NA/NR	Y	Y	10	Good

27	Van den Berg et al., 2016	Y	Y	N	Y	N	N	Y	N	Y	N	Y	NA/NR	NA/NR	Y	7	Fair
28	Wood et al., 2020	Y	Y	NA/NR	Y	N	N	Y	Y	Y	N	Y	NA/NR	NA/NR	N	7	Fair
29	Younan et al., 2016	Y	Y	NA/NR	Y	N	Y	Y	Y	Y	Y	Y	NA/NR	Y	Y	11	Good

* Assessment Items from the Quality Assessment Tool for Observational Cohort and Cross-Sectional Studies by the National Heart, Lung, and Blood Institute.

1. Was the research question or objective in this paper clearly stated?
2. Was the study population clearly specified and defined?
3. Was the participation rate of eligible persons at least 50%?
4. Were all the subjects selected or recruited from the same or similar populations? Were inclusion and exclusion criteria for being in the study prespecified and applied uniformly to all participants?
5. Was a sample size justification, power description, or variance and effect estimates provided?
6. For the analyses in this paper, were the exposure(s) of interest measured prior to the outcome(s) being measured?
7. Was the timeframe sufficient so that one could reasonably expect to see an association between exposure and outcome if it existed?
8. For exposures that can vary in amount or level, did the study examine different levels of the exposure as related to the outcome?
9. Were the exposure measures (independent variables) clearly defined, valid, reliable, and implemented consistently across all study participants?
10. Was the exposure(s) assessed more than once over time?
11. Were the outcome measures (dependent variables) clearly defined, valid, reliable, and implemented consistently across all study participants?
12. Were the outcome assessors blinded to the exposure status of participants?
13. Was loss to follow-up after baseline 20% or less?
14. Were key potential confounding variables measured and adjusted statistically for their impact on the relationship between exposure(s) and outcome(s)?

Supplementary Material III.

Table S2. Qualitative Studies Identified and Reviewed.

	Author-year	Country	Population	Sample size	Methods
1 & 2	Bell et al., 2015<br>Bell et al., 2017	UK	Adults (25–85 yr) reside in coastal towns	33	Activity maps, GPS tracking, geo-narrative interviews, go-along interviews
3	Diduck et al., 2020	Canada	Adults (in some cases couples/family) residents engaged in home gardening	42	Semi-structured interviews
4	Husser et al., 2020	US	Older women (71–91 yr) in rural areas	34	In-depth interviews (four times during a five-year period)
5	Lloyd et al., 2008	Australia	Teenage girls (14–18 yr)	11	Semi-structured interviews
6	MacIntyre et al., 2019	Multi-country	Extreme sport athletes	8	Semi-structured interviews
7	Milligan & Bingley, 2007	US	Young adults (16–21 yr)	16	Focus group, workshop sessions (e.g., walk, craft session, sand play, modeling), in-depth interview
8	Pace & Walker, 2020	Canada	Older adults (50+ yr) Southern Inuit of Nunatu Kavut	14	Photovoice

9	Weimann et al., 2019	Sweden	Adults (18–70 yr)	16	Interviews
